# Supplementary material for: CLAMP and Zelda function together to promote Drosophila zygotic genome activation
Source: eLife. 2021 Aug 3;10:e69937. doi: 10.7554/eLife.69937 (PMC8367384; doi:10.7554/eLife.69937)
Supplement: Figure 1—source data 1. [file elife-69937-fig1-data1.zip › Figure 1-Source Data 1/Figure 1 Sources Data Captions.docx]

Figure 1- Source Data

1. Western blot of CLAMP and reference control Actin in MTD, *clamp-i* and *zld-i* embryos in 0-2hr and 2-4hr embryos. MTD: MTD-Gal4 line. *clamp-i:* MTD-Gal4-*clamp* mRNAi line, *zld-i:* MTD-Gal4-*zld* mRNAi line.
2. Western blot of ZLD and reference control Actin in MTD, *clamp-i* and *zld-i* embryos in 0-2hr and 2-4hr embryos. MTD: MTD-Gal4 line. *clamp-i:* MTD-Gal4-*clamp* mRNAi line, *zld-i:* MTD-Gal4-*zld* mRNAi line.
3. Electrophoretic mobility shift assay (EMSA) showing the binding of increasing amounts of CLAMP DNA-binding domain (DBD) fused to MBP to 5C2 naked DNA or 5C2 *in vitro* reconstituted nucleosomes (NUC). Concentrations (uM) of CLAMP DBD increase from left to right.
4. EMSA showing the binding of increasing amounts of full-length (FL) CLAMP (fused to MBP) to 5C2 DNA or 5C2 nucleosomes (NUC). Concentrations (uM) of CLAMP FL increase from left to right.
