# Supplementary figures and images for: CLAMP and Zelda function together to promote Drosophila zygotic genome activation
Source: eLife. 2021 Aug 3;10:e69937. doi: 10.7554/eLife.69937 (PMC8367384; doi:10.7554/eLife.69937)

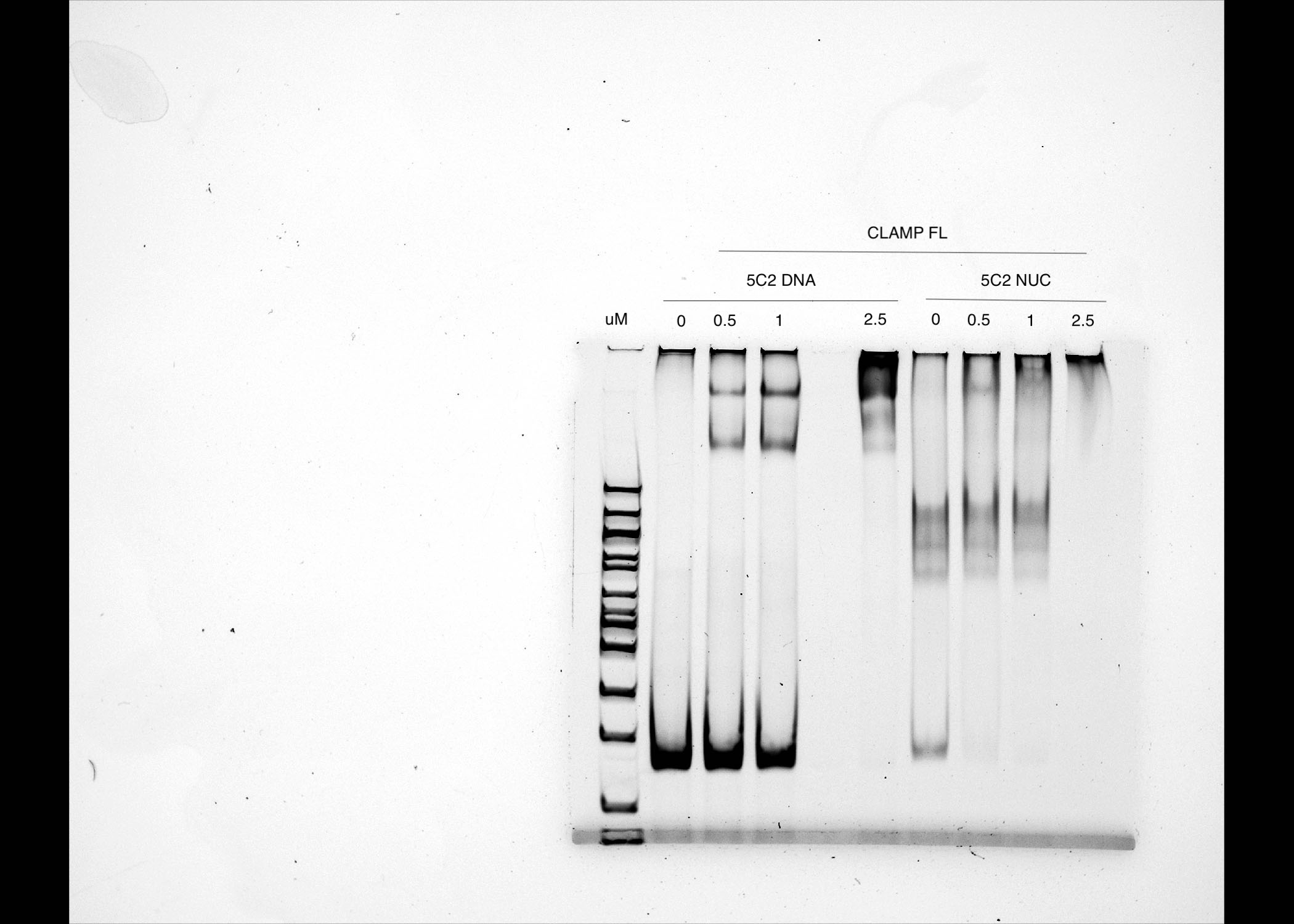

Supplement: Figure 1—source data 1. [file elife-69937-fig1-data1.zip › Figure 1-Source Data 1/Fig1 Source data 4.jpg]

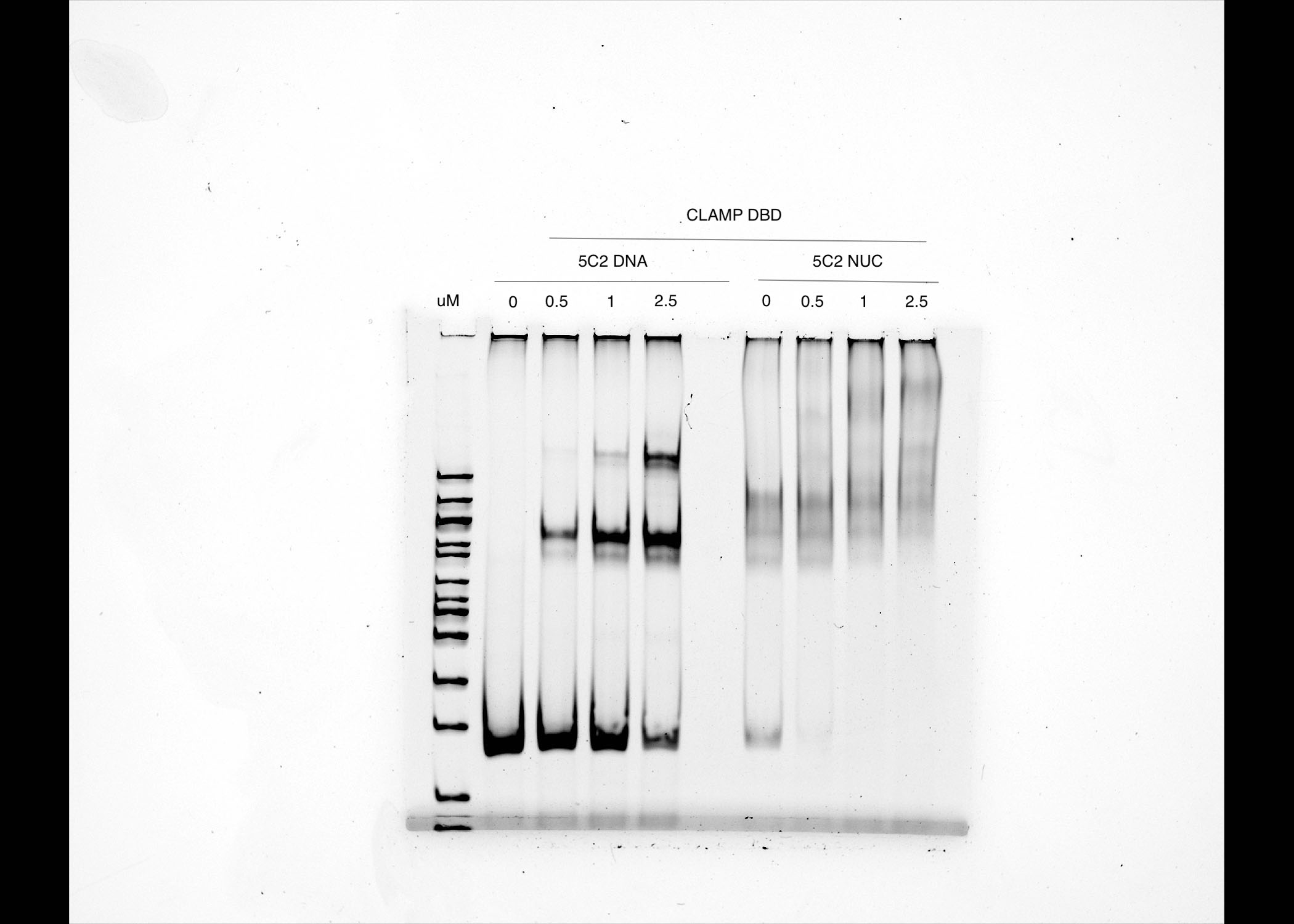

Supplement: Figure 1—source data 1. [file elife-69937-fig1-data1.zip › Figure 1-Source Data 1/Fig1 Source data 3.jpg]

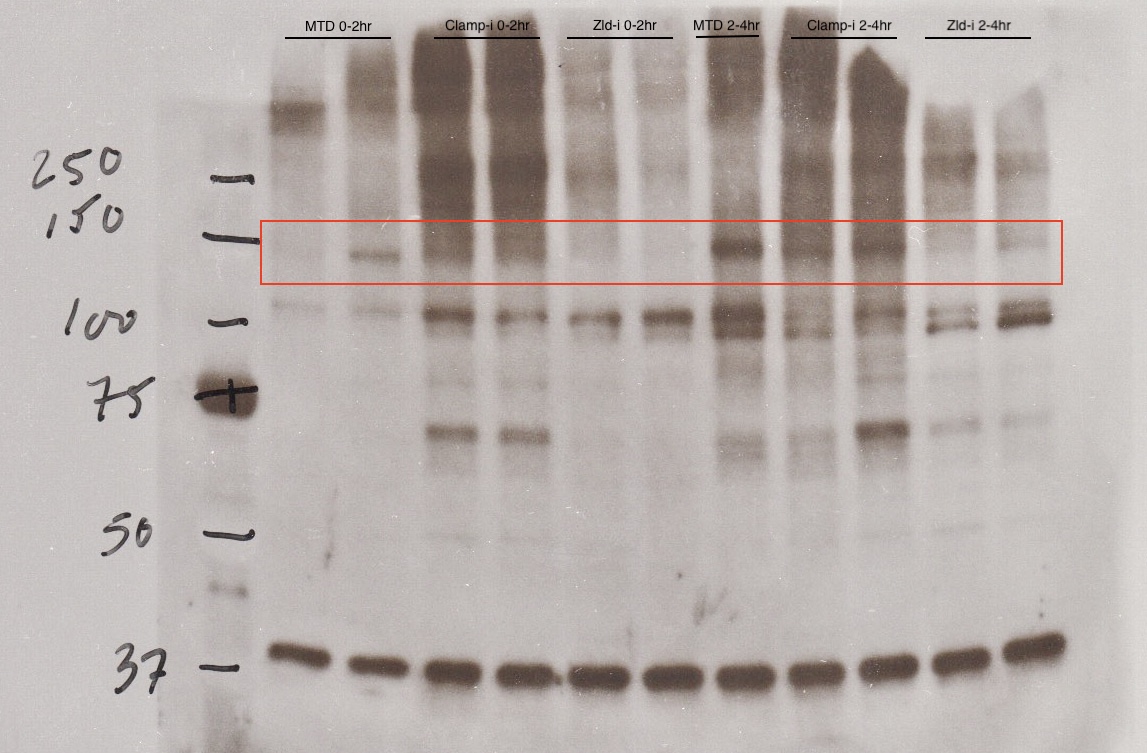

Supplement: Figure 1—source data 1. [file elife-69937-fig1-data1.zip › Figure 1-Source Data 1/Fig1 Source data 2.jpg]

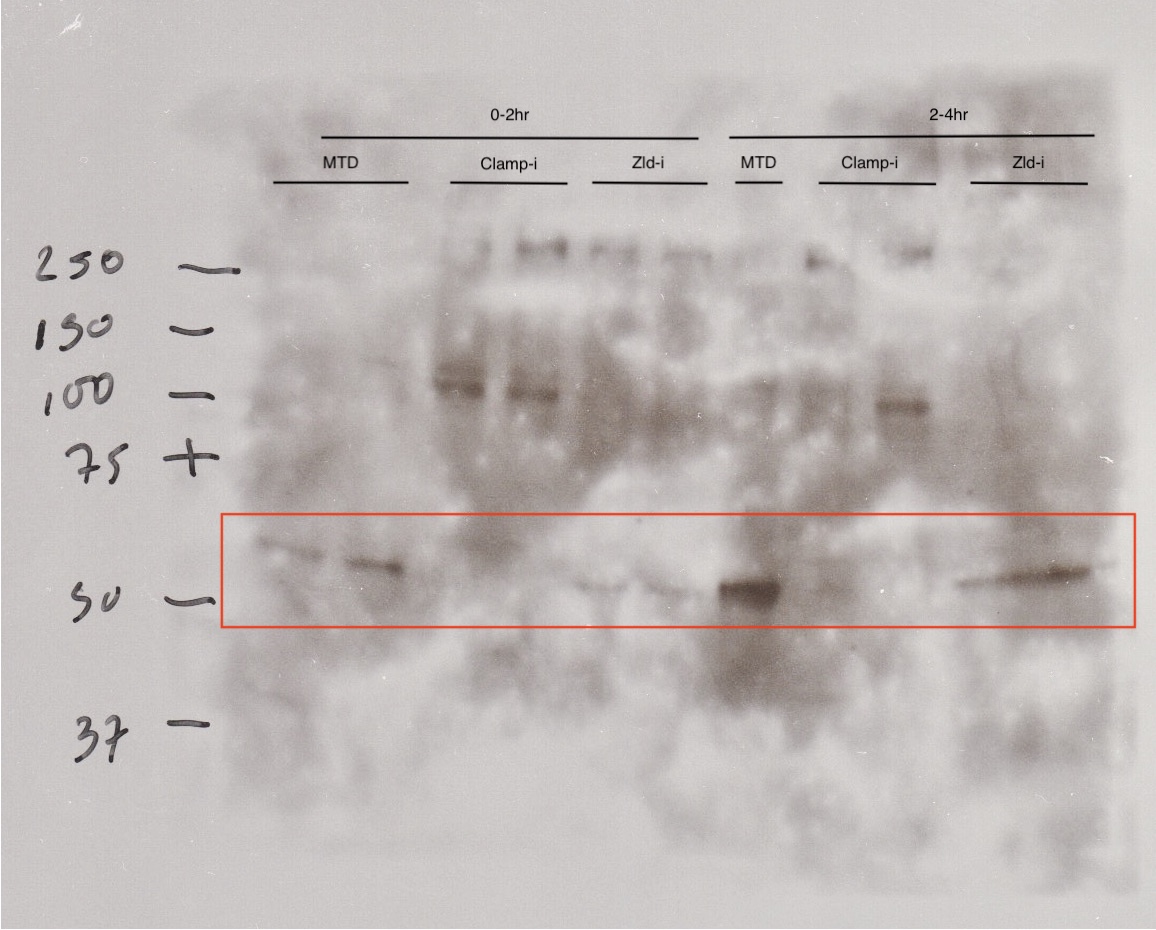

Supplement: Figure 1—source data 1. [file elife-69937-fig1-data1.zip › Figure 1-Source Data 1/Fig1 Source data 1.jpg]
