# Supplementary material for: CLAMP and Zelda function together to promote Drosophila zygotic genome activation
Source: eLife. 2021 Aug 3;10:e69937. doi: 10.7554/eLife.69937 (PMC8367384; doi:10.7554/eLife.69937)
Supplement: Transparent reporting form [file elife-69937-transrepform.pdf]

eLIFE
1st Floor
24 Hills Road
Cambridge CB2 1JP, UK
P 01223 855340
W elifesciences.org
T @elife

eLife's transparent reporting form

We encourage authors to provide detailed information *within their submission* to facilitate the interpretation and replication of experiments. Authors can upload supporting documentation to indicate the use of appropriate reporting guidelines for health-related research (see EQUATOR Network), life science research (see the BioSharing Information Resource), or the ARRIVE guidelines for reporting work involving animal research. Where applicable, authors should refer to any relevant reporting standards documents in this form.

If you have any questions, please consult our Journal Policies and/or contact us: editorial@elifesciences.org.

Sample-size estimation

- You should state whether an appropriate sample size was computed when the study was being designed
- You should state the statistical method of sample size computation and any required assumptions
- If no explicit power analysis was used, you should describe how you decided what sample (replicate) size (number) to use

Please outline where this information can be found within the submission (e.g., sections or figure legends), or explain why this information doesn't apply to your submission:

Sample size was not computed as a part of experimental design. Multiple independent samples were collected to achieve satisfactory sequencing depth and reproducibility.

Sample sizes for ATAC-seq were 10 embryos, as stated in materials and methods. Sample sizes for ChIP-seq were 100 embryos, as indicated in materials and methods.

Replicates

- You should report how often each experiment was performed
- You should include a definition of biological versus technical replication
- The data obtained should be provided and sufficient information should be provided to indicate the number of independent biological and/or technical replicates
- If you encountered any outliers, you should describe how these were handled
- Criteria for exclusion/inclusion of data should be clearly stated
- High-throughput sequence data should be uploaded before submission, with a private link for reviewers provided (these are available from both GEO and ArrayExpress)

Please outline where this information can be found within the submission (e.g., sections or figure legends), or explain why this information doesn't apply to your submission:

eLIFE
1st Floor
24 Hills Road
Cambridge CB2 1JP, UK
P 01223 855340
W elifesciences.org
T @elife

No technical replicates were used in this study. All replicates discussed are biological.
To quantify relative protein levels from immunoblots and mRNA level from qRT-PCR, two biological replicates were used for each genotype, as indicated in materials and methods.
For ATAC-seq, we performed three biological replicates for each genotype (n=2) and time point (n=2), total of 12 samples, as indicated in materials and methods.
For ChIP-seq, we performed three biological ChIP replicates for each protein (n=2), genotype (n=3) and time point (n=2). In total of 36 libraries in materials and methods.
ChIP-seq peaks were called on all 3 biological replicates, and used MSPC to obtain consensus peaks from 3 replicates as described in materials and methods.

Statistical reporting

- Statistical analysis methods should be described and justified
- Raw data should be presented in figures whenever informative to do so (typically when N per group is less than 10)
- For each experiment, you should identify the statistical tests used, exact values of N, definitions of center, methods of multiple test correction, and dispersion and precision measures (e.g., mean, median, SD, SEM, confidence intervals; and, for the major substantive results, a measure of effect size (e.g., Pearson's r, Cohen's d)
- Report exact p-values wherever possible alongside the summary statistics and 95% confidence intervals. These should be reported for all key questions and not only when the p-value is less than 0.05.

Please outline where this information can be found within the submission (e.g., sections or figure legends), or explain why this information doesn't apply to your submission:

ATAC-seq peaks were classified by determining statistically significant differences in sequencing read counts using DiffBind software as described on page 12 Figure 2A. This software was also used to identify significantly altered peaks between ChIP-seq datasets, as described in materials and methods.
Statistical tests for p-values are described in the Results section and indicated in materials and methods.

(For large datasets, or papers with a very large number of statistical tests, you may upload a single table file with tests, Ns, etc., with reference to sections in the manuscript.)

Group allocation

- Indicate how samples were allocated into experimental groups (in the case of clinical studies, please specify allocation to treatment method); if randomization was used, please also state if restricted randomization was applied
- Indicate if masking was used during group allocation, data collection and/or data analysis

Please outline where this information can be found within the submission (e.g., sections or figure legends), or explain why this information doesn't apply to your submission:

Embryo samples were randomly collected for each time points: 0-2hr and 2-4hr post egg lay.

Additional data files ("source data")

- We encourage you to upload relevant additional data files, such as numerical data that are represented as a graph in a figure, or as a summary table

eLife Sciences Publications, Ltd is a limited liability non-profit non-stock corporation incorporated in the State of Delaware, USA, with company number 5030732, and is registered in the UK with company number FC030576 and branch number BR015634 at the address 1st Floor, 24 Hills Road, Cambridge CB2 1JP | August 2014

- Where provided, these should be in the most useful format, and they can be uploaded as “Source data” files linked to a main figure or table
- Include model definition files including the full list of parameters used
- Include code used for data analysis (e.g., R, MatLab)
- Avoid stating that data files are “available upon request”

Please indicate the figures or tables for which source data files have been provided:

Data accessibility for reviewer
ATAC-seq (GSE152596) and ChIP-seq (GSE152598) are under GEO SuperSeries GSE152613. To review GEO accession GSE152613:
Go to https://www.ncbi.nlm.nih.gov/geo/query/acc.cgi?acc=GSE152613
Enter token ihevsmiqnxexrod into the box

High throughput sequencing data analysis was performed using available command-line tools and R packages as indicated clearly in the Results and Material and Methods.
